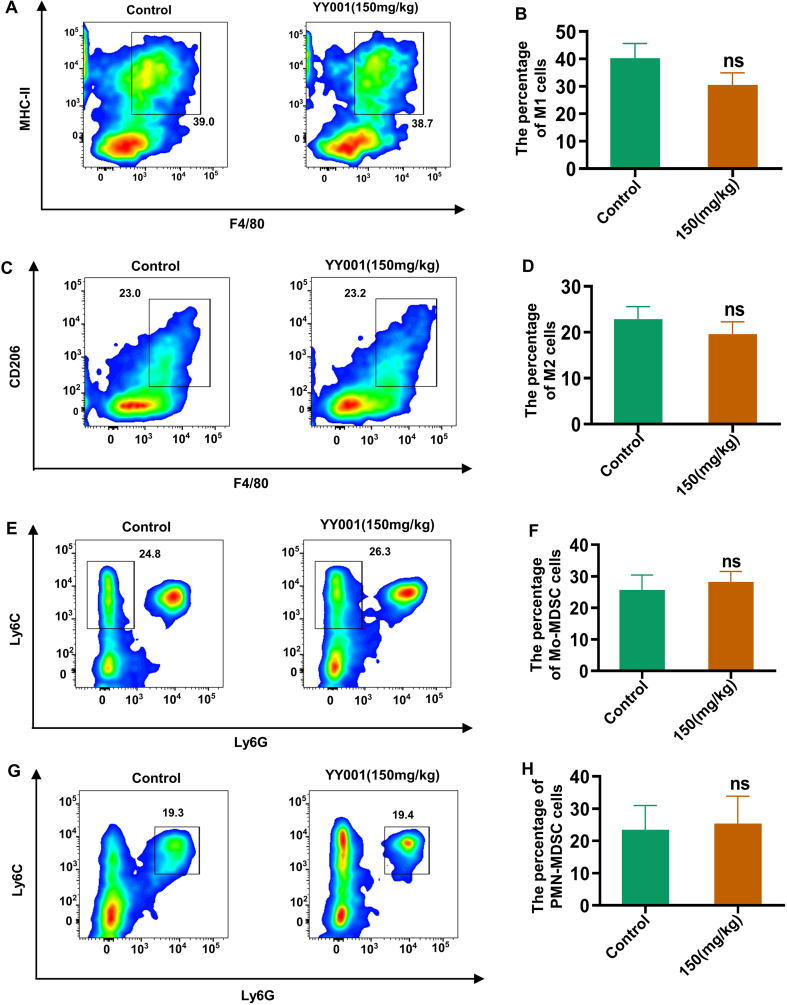# Remodeling the immune microenvironment for gastric cancer therapy through antagonism of prostaglandin E2 receptor 4

**DOI:** 10.1016/j.gendis.2023.101164

**Published:** 2023-11-10

**Authors:** Mengmeng Guo, Pan Hu, Jiayi Xie, Kefu Tang, Shixiu Hu, Jialiang Sun, Yundong He, Jing Li, Weiqiang Lu, Huirong Liu, Mingyao Liu, Zhengfang Yi, Shihong Peng

**Affiliations:** a Shanghai Key Laboratory of Regulatory Biology, School of Life Sciences, East China Normal University, Shanghai 200241, China; b Prenatal Diagnosis Center, Department of Clinical Laboratory, Changning Maternity and Infant Health Hospital, East China Normal University, Shanghai 200051, China; c Key Laboratory of Acupuncture and Immunological Effects, Shanghai University of Traditional Chinese Medicine, Shanghai 201203, China; d Fengxian Hospital Affiliated to Southern Medical University, Shanghai 201400, China; e Shanghai Yuyao Biotech Co., Ltd., Shanghai 200241, China

**Keywords:** EP4, Immunomodulation, Synergy, Tumor microenvironment, YY001

## Abstract

Gastric cancer is highly prevalent among digestive tract tumors. Due to the intricate nature of the gastric cancer immune microenvironment, there is currently no effective treatment available for advanced gastric cancer. However, there is promising potential for immunotherapy targeting the prostaglandin E2 receptor subtype 4 (EP4) in gastric cancer. In our previous study, we identified a novel small molecule EP4 receptor antagonist called YY001. Treatment with YY001 alone demonstrated a significant reduction in gastric cancer growth and inhibited tumor metastasis to the lungs in a mouse model. Furthermore, administration of YY001 stimulated a robust immune response within the tumor microenvironment, characterized by increased infiltration of antigen-presenting cells, T cells, and M1 macrophages. Additionally, our research revealed that YY001 exhibited remarkable synergistic effects when combined with the PD-1 antibody and the clinically targeted drug apatinib, rather than fluorouracil. These findings suggest that YY001 holds great promise as a potential therapeutic strategy for gastric cancer, whether used as a standalone treatment or in combination with other drugs.

## Introduction

Gastric cancer ranks fourth as the leading cause of cancer-related deaths worldwide.^1^ The development of gastric cancer involves a complex and multi-stage process, with various biological factors at play.^2^^,^^3^ Due to the lack of specific symptoms and low rates of early diagnosis, most patients are typically diagnosed at an advanced stage of the disease.^4^^,^^5^ Traditional treatments like chemotherapy and radiation have limited effectiveness against advanced gastric cancer, resulting in poor prognoses.^6^^,^^7^ While targeted drugs such as trastuzumab, lapatinib, cetuximab, and bevacizumab have been investigated in clinical research, their overall outcomes have been disappointing.^7^^,^^8^ Recently, certain immunotherapies have shown promise in melanoma, Hodgkin's lymphoma, and non-small cell lung cancer.^9^, ^10^, ^11^ However, immunotherapies like ipilimumab and pembrolizumab have not achieved the desired effects in gastric cancer.^12^ Further research on the immunosuppressive effects in gastric cancer may help optimize immunotherapy approaches.

Cancer initiation and progression are significantly influenced by the tumor microenvironment. It consists of neighboring blood vessels, signaling molecules, non-cancerous cells, and extracellular matrix.^13^^,^^14^ Multiple immune cell types are found in the tumor microenvironment, including myeloid-derived suppressor cells (MDSCs), dendritic cells, tumor-associated macrophages, T cells, and natural killer cells.^15^ Upon recognizing tumor antigens, tumor-reactive effector T cells, which are known as tumor-specific T cells, can secrete potent effector cytokines such as tumor necrosis factor-α and interferon-γ.^16^^,^^17^ Dendritic cells can promote the expression of Th1 and cytotoxic immune co-stimulatory molecules, such as interleukin (IL)-12 and IL-23.^18^^,^^19^ MDSCs primarily exhibit immune suppression and inhibit the activation of various immune cells through the production of inducible nitric oxide synthase and reactive oxygen species.^20^, ^21^, ^22^ Tumor-associated macrophages can be classified into two types: M1-type macrophages that secrete pro-inflammatory factors like tumor necrosis factor-α, interferon-γ, and IL-β, and M2-type macrophages that secrete anti-inflammatory factors such as IL-4, TGF-β, and epithelial growth factors, promoting tumor proliferation and migration.^23^, ^24^, ^25^

Prostaglandin E2 (PGE2) is the main product of arachidonic acid metabolism mediated by cyclooxygenase and PGE synthase.^26^, ^27^, ^28^, ^29^ PGE2 acts on G protein-coupled receptors, including four subtypes known as EP1-4, thereby regulating signal transduction pathways.^30^^,^^31^ EP4, in particular, can activate various intracellular signaling pathways, such as the protein kinase A pathway, adenylate cyclase stimulation, and the cAMP/PKA signaling pathway, thereby promoting the invasive behavior of cancer cells.^32^, ^33^, ^34^ EP4 also plays a significant role in immunosuppression, inhibiting natural killer cell functions in breast cancer,^35^ recruiting regulatory T cells, and suppressing T cell activation.^36^^,^^37^ Preclinical models have shown promising results with EP4 inhibitors in several cancers, including breast cancer and prostate cancer.^38^^,^^39^

EP4 promotes cancer cell migration and invasion and exerts immunosuppressive effects.^33^^,^^37^^,^^40^ In our current study, we investigated a novel EP4 receptor antagonist called YY001, which demonstrated the ability to inhibit MDSC differentiation and increase T-cell infiltration. When combined with PD-1 antibody, in some instances, YY001 not only suppressed tumor growth but also resulted in complete tumor regression. These findings suggest that YY001 has the potential to remodel the tumor immune microenvironment in gastric cancer.

Overall, gastric cancer is a challenging disease due to its multifactorial nature, late diagnosis, and limited treatment options for advanced stages. While targeted drugs and immunotherapies have shown promise in other cancer types, their effectiveness in gastric cancer remains limited. However, further research on immunosuppressive mechanisms and the development of novel therapeutic approaches, such as EP4 antagonists like YY001, may help optimize immunotherapy strategies for gastric cancer patients.

## Materials and methods

### Cell lines

The mouse gastric cancer cell line MFC was obtained from the China Infrastructure of Cell Line Resource, while the mouse melanoma cell line B16F10 was acquired from the Chinese Academy of Sciences Type Culture Collection Cell Bank. These cell lines were cultured in a growth medium recommended by the vendor, which was supplemented with 10% (v/v) fetal bovine serum and 1% antibiotics (penicillin and streptomycin). The cultures were maintained at 37 °C with 5% CO_2_.

### Subcutaneous gastric cancer model

Six-to-eight-week-old 615 mice were obtained from the Animal Center of East China Normal University, and their welfare was monitored in compliance with the guidelines of the Animal Investigation Committee at the Institute of Biomedical Sciences, East China Normal University. MFC cells were adjusted to a concentration of 1 × 10^7^ cells/mL in a phosphate-buffered saline solution. Subsequently, 100 μL of the MFC cell suspension was injected into the right subcutaneous region of the mice's backs. Tumor-bearing mice were randomly divided into groups, and YY001 (synthesized internally in the laboratory; its structure is described in our previous study^41^) was dissolved in sterile water containing 0.5% carboxymethylcellulose. It was administered orally once daily. Two different doses of YY001, 75 mg/kg or 150 mg/kg, were administered. Opdivo (Anti-PD-1, BioXCell, USA) was administered intraperitoneally at a dose of 10 μg per administration, while 5-Fu was administered at a dose of 20 mg/kg once a day. Apatinib was administered via gavage at a dose of 75 mg/kg once a day. In the combined treatment group, YY001 was administered orally at a daily dose of 75 mg/kg, along with anti-PD-1 at a dose of 20 μg per administration or 5-Fu at a dose of 20 mg/kg, or apatinib at a dose of 75 mg/kg. Tumor size was calculated using the formula: length × width^2^ × 0.52. At the end of the treatment period, the mice were euthanized, and the tumors were removed, photographed, weighed, and fixed for subsequent validation.

### Gastric cancer lung metastasis model

MFC cells were utilized to establish a lung metastasis model through tail vein injection. The mice were randomly divided into three groups and orally administered either 0.5% carboxymethylcellulose at a dose of 75 mg/kg or 150 mg/kg of YY001 once daily. Daily observations of the mice were conducted, and their body weight was measured every three days. After the experiment, the mice were euthanized, and the lung tissue was dissected for further experimentation.

### qPCR

Total RNA was extracted from tumor tissues using the TRIzol method. Subsequently, 1 μg of RNA was reverse transcribed into cDNA using the PrimeScript cDNA synthesis kit. SYBR Green-based quantitative PCR assays were conducted using mouse primers specific to CXCL9, CXCL10, CXCL11, interferon-γ, tumor necrosis factor-α, GZMB, and Perforin. The primer sequences can be found in Table S1. The expression levels of the target gene mRNA were normalized to β-actin using the ΔΔCt method.

### Immunofluorescence analysis

The MFC tumor tissues were embedded, sectioned, and dewaxed. Following antigen repair, they were covered and incubated with specific primary antibodies. Subsequently, they were washed with phosphate-buffered saline solution with Tween® 20 and incubated with secondary antibodies at room temperature, while being shielded from light. After thorough washing, DAPI was applied drop by drop to stain the nuclei, and an anti-quenching agent was added to prevent fluorescence quenching. Finally, the sections were sealed for observation.

### *In vitro* study of dendritic cells and MDSCs from mouse bone marrow cells

Under aseptic conditions, the tibia and femur bones of 615 mice were obtained. The bone marrow was flushed, and single-cell suspensions were prepared. Following the removal of erythrocytes, the cells were inoculated into 6-well plates and cultured in a medium containing RPMI1640, granulocyte-macrophage colony-stimulating factor, and IL-4. YY001 or E7046 was added to the medium in the presence or absence of PGE2 (Cayman Chemical, USA). The cells were then incubated at 37 °C in a 5% CO_2_ incubator. Subsequently, the cells were collected and analyzed using flow cytometry (BD LSRFortessa).

### Detection of expression of immunosuppressive molecules in MDSCs

Mouse bone marrow-derived cells were cultured in RPMI1640 medium supplemented with granulocyte-macrophage colony-stimulating factor and IL-6. To evaluate the effects of treatment, PGE2, YY001, or E7046 were added. After treatment, MDSCs were collected, and total RNA was extracted using TRIzol. Quantitative PCR was performed to measure the transcriptional expression of immunosuppressive molecules such as Arg1, inducible nitric oxide synthase, COX2, and others. The primer sequences can be found in Table S1.

### Flow cytometry analysis

The tumor tissue was isolated, fragmented into small pieces, and enzymatically digested using collagenase in a serum-free RPMI1640 medium. Subsequently, the digested tumor tissue underwent filtration through a cell filter to obtain a homogeneous single-cell suspension. To identify cell surface antigens, flow antibodies were used for staining, and the expression of various cellular markers was analyzed using a flow cytometer called BD LSRFortessa. The antibody information can be found in Table S2.

### Statistical analysis

All experimental data were collected from three or more replicates. Statistical analysis was conducted using the student *t*-test, where a *P*-value less than 0.05 was deemed statistically significant. The analysis was performed using GraphPad Prism 8. Additionally, a one-way ANOVA was employed to compare the control group with the experimental group.

## Results

### YY001 effectively suppresses the growth and metastasis of gastric cancer

**Figure 1:**
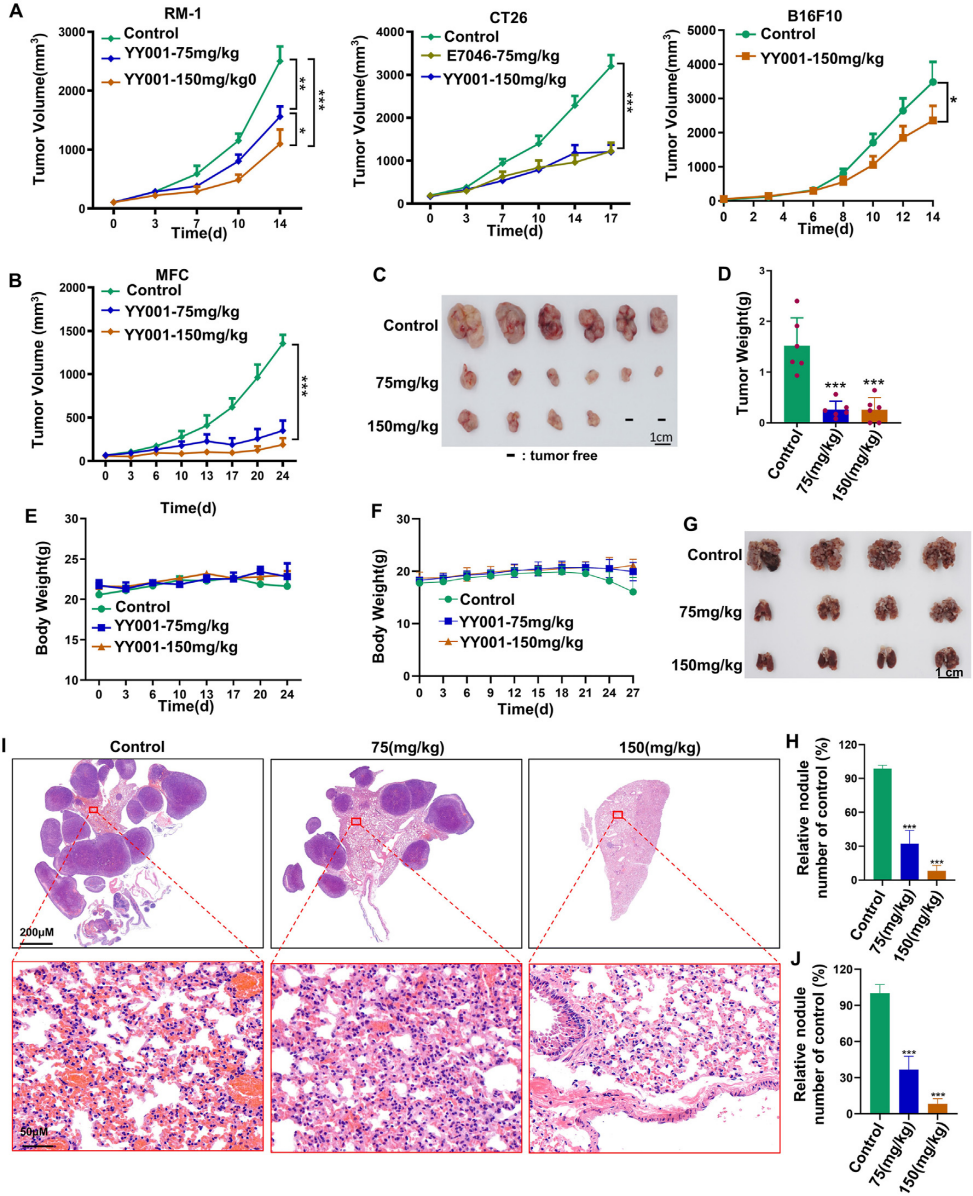
YY001 inhibits the tumor growth and metastasis of gastric cancer. **(A)** The anti-tumor activity of YY001 was evaluated in different tumor models (prostate cancer, melanoma, and colon cancer). **(B–E)** Evaluation of the anti-tumor growth activity of YY001 in an MFC immune-sound mouse subcutaneous growth model of gastric cancer. (B) Tumor growth curve. (C) Tumor peeling white light plot. (D) Tumor weight statistics. (E) Mouse weight change curve. **(F–H)** Evaluation of anti-tumor metastatic activity of YY001 in an immunocompetent mouse tail vein lung metastasis model. (F) Body weight change curve of mice. (G) White light map of stripped lung tissue. (H) Statistical analysis of mouse lung tumors. **(I)** Hematoxylin and eosin detection of tumor cells in lung tissue of mice in a lung metastasis model. **(J)** Statistical analysis of tumorigenic lesions after hematoxylin and eosin staining. ∗*P* < 0.05; ∗∗*P* < 0.01; ∗∗∗*P* < 0.001.

The EP4 receptor antagonist competes with PGE2 for binding to EP4 receptors, inhibiting the development and progression of inflammation. This, in turn, reverses the immunosuppressive effect produced by PGE2 and enhances immune function, leading to the inhibition of tumor growth. Therefore, it represents a potential target for tumor immunotherapy. We evaluated the anti-tumor activity of YY001 in animal models using various immunotherapy evaluation models, including colon cancer, melanoma, gastric cancer, and prostate cancer, by selecting mouse tumor cell lines in immunocompetent mice.^26^ In these models, we preliminarily assessed the anti-tumor activity of YY001 in different types of tumors. We discovered that YY001 monotherapy exhibited a more effective tumor suppressive effect in colorectal and gastric cancer. The tumor inhibition rates reached 62.4% and 74.2% (Fig. 1A and B) respectively. Additionally, gastric cancer growth inhibition by YY001 showed dose-dependent effects as observed through peeling (Fig. 1C) and weighing the tumor tissues (Fig. 1D). These results indicated that YY001 had a significant inhibitory effect on MFC tumor growth, with approximately 30% complete tumor elimination at higher doses (Fig. 1C). Throughout the treatment, no significant weight changes were observed, indicating that YY001 is safe and well-tolerated (Fig. 1E). Furthermore, we evaluated the effect of YY001 on tumor metastasis through a tail vein lung metastasis model. On day 21 of administration, control mice exhibited a slight decrease in body weight, leading us to terminate the experiment on day 27 (Fig. 1F). Upon dissecting the lungs of the mice, we observed numerous metastatic tumors in the control group, along with a significant increase in lung volume. However, in the YY001 treatment group, the number of lung tumors decreased significantly with increasing doses, and the lung morphology appeared more normal (Fig. 1G). By counting the tumors in the lungs of the mice, it is more obvious to see a significant reduction in tumors that metastasize to the lungs after YY001 treatment (Fig. 1H). Hematoxylin and eosin stains of lung tissue from mice in the lung metastasis model revealed that the control group had more cancer cells and eosinophilic tumorigenic lesions (Fig. 1I). A statistical analysis of eosinophilic tumorigenic lesions in lung tissues after hematoxylin and eosin staining yielded the same results (Fig. 1J). These findings indicate that YY001 treatment significantly inhibits the growth and metastasis of gastric cancer.

**Figure S1:**
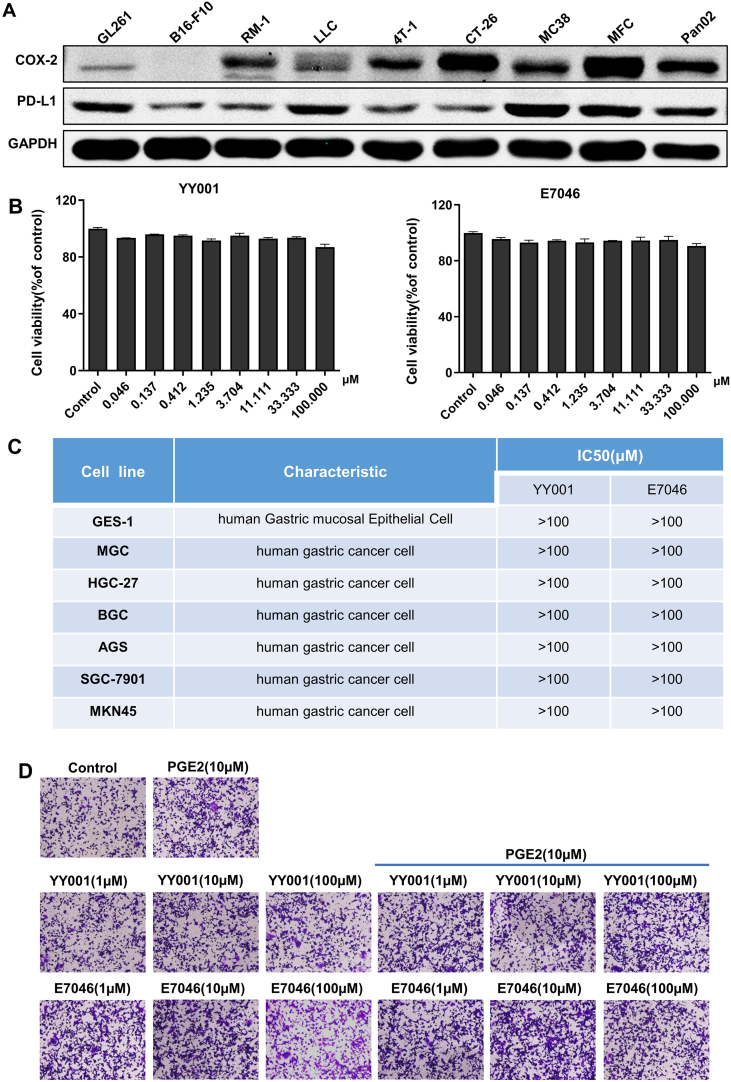


Subsequently, we performed western blot validation on COX-2/PGE2/EP4 pathway-related proteins in different tumor cells and found that the key enzyme COX-2 was highly expressed in gastric cancer, leading to abnormal pathway activation (Fig. S1A). *In vitro* treatment with YY001 did not affect the cellular activity of the MFC cell line (Fig. S1B). Moreover, testing different gastric cancer cell lines revealed that the IC50 after YY001 treatment exceeded 100 μM (Fig. S1C). Transwell assay experiments demonstrated that YY001 did not affect the migration of mouse gastric cancer cells *in vitro* (Fig. S1D). These results suggest that YY001 does not exert its tumor-inhibiting effect through direct action on tumor cells.

### YY001 modulates T cell, MDSC, and antigen-presenting cell infiltration and function

**Figure 2:**
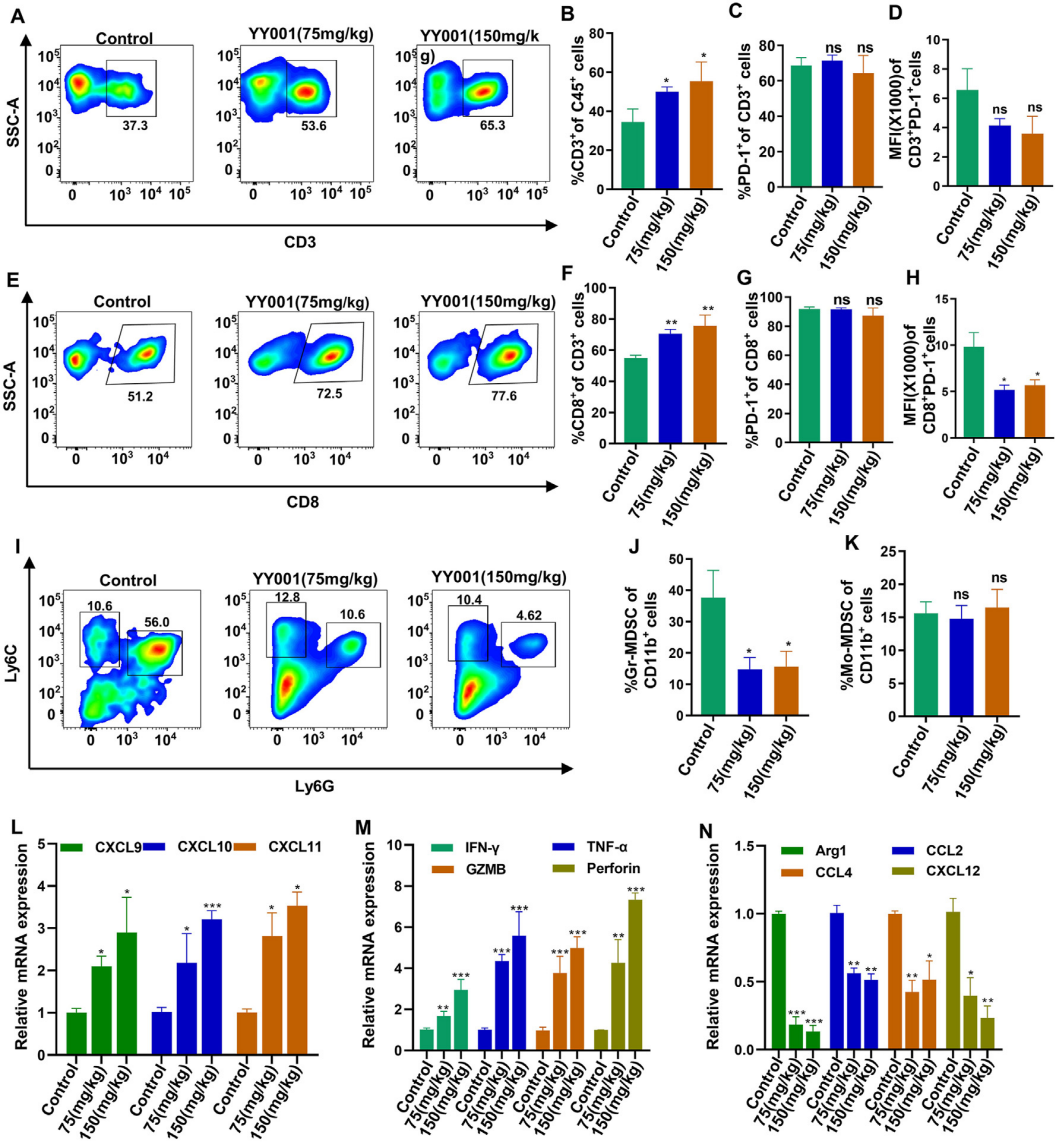
YY001 regulates the infiltration and function of MDSCs and T cells. **(A–D)** Infiltration of CD3 cells in the tumor microenvironment by flow analysis. (A) Flow analysis. (B) Statistical result. (C) Statistical analysis of PD-1 expression. (D) Statistical analysis of the mean fluorescence intensity of PD-1. **(E–H)** Flow analysis of CD8 cell infiltration in the tumor microenvironment. (E) Flow analysis. (F) Statistical result. (G) Statistical result of PD-1 expression. (H) Statistical analysis of the mean fluorescence intensity of PD-1. **(I–K)** Infiltration of MDSCs in the tumor microenvironment by flow analysis. (I) Flow analysis. (J) Granulocyte MDSC (Gr-MDSC) statistics. (K) Mononuclear MDSC (Mo-MDSC) statistics. **(L)** Quantitative PCR analysis of the expression of cytokines associated with T cell recruitment. **(M)** Quantitative PCR to detect the expression of molecules associated with T cell function. **(N)** Detection of MDSC expression by quantitative PCR. ∗*P* < 0.05; ∗∗*P* < 0.01; ∗∗∗*P* < 0.01; ns, not significant.

To investigate the tumor suppressive effect of YY001, tumor tissues were isolated from mice and digested into single-cell suspensions. Flow cytometry analysis was conducted to examine the presence of major immune cell groups. Initially, T cells were the focus of the investigation due to their crucial role in the immune system's ability to eliminate tumors. The results revealed a significant increase in the infiltration of CD3^+^ T cells (Fig. 2A and B) and CD8^+^ T cells (Fig. 2E and F) into the tumor immune microenvironment. In addition, we conducted a statistical analysis of PD-1 expression in T cells. Although the percentage of PD-1 expression did not significantly decrease compared with the control group (Fig. 2C, G), we observed a decrease in the fluorescence intensity of PD-1. Therefore, a statistical analysis was performed on the mean fluorescence intensity, which showed a significant reduction (Fig. 2D, H). This indicates that YY001 can reduce the expression of PD-1 in T cells. Next, the investigation turned to MDSCs, which have a suppressive effect on T-cell function (Fig. 2I). The infiltration of MDSCs was significantly reduced after YY001 treatment, with granulocytes being the main class of cells that exhibited a reduction (Fig. 2J and K). Subsequently, the secretion of cytokines related to T cell recruitment and the expression of molecules associated with T cell function was assessed using quantitative PCR. Following YY001 treatment, the tumor tissue exhibited increased secretion of cytokines such as CXCL9, CXCL10, and CXCL11, which contribute to the recruitment of more T cells (Fig. 2L). The expression of tumor cell-killing factors such as interferon-γ, tumor necrosis factor-α, and granzyme B by T cells was significantly increased, resulting in enhanced tumor cell killing (Fig. 2M). The expression of MDSC-related markers, including Arg1, CCL2, CCL4, and CCL12, was significantly reduced (Fig. 2N). These findings suggest that YY001 treatment effectively increases T cell infiltration in tumor tissue and enhances the expression of tumor-killing factors, thereby exerting an inhibitory effect on tumors.

**Figure 3:**
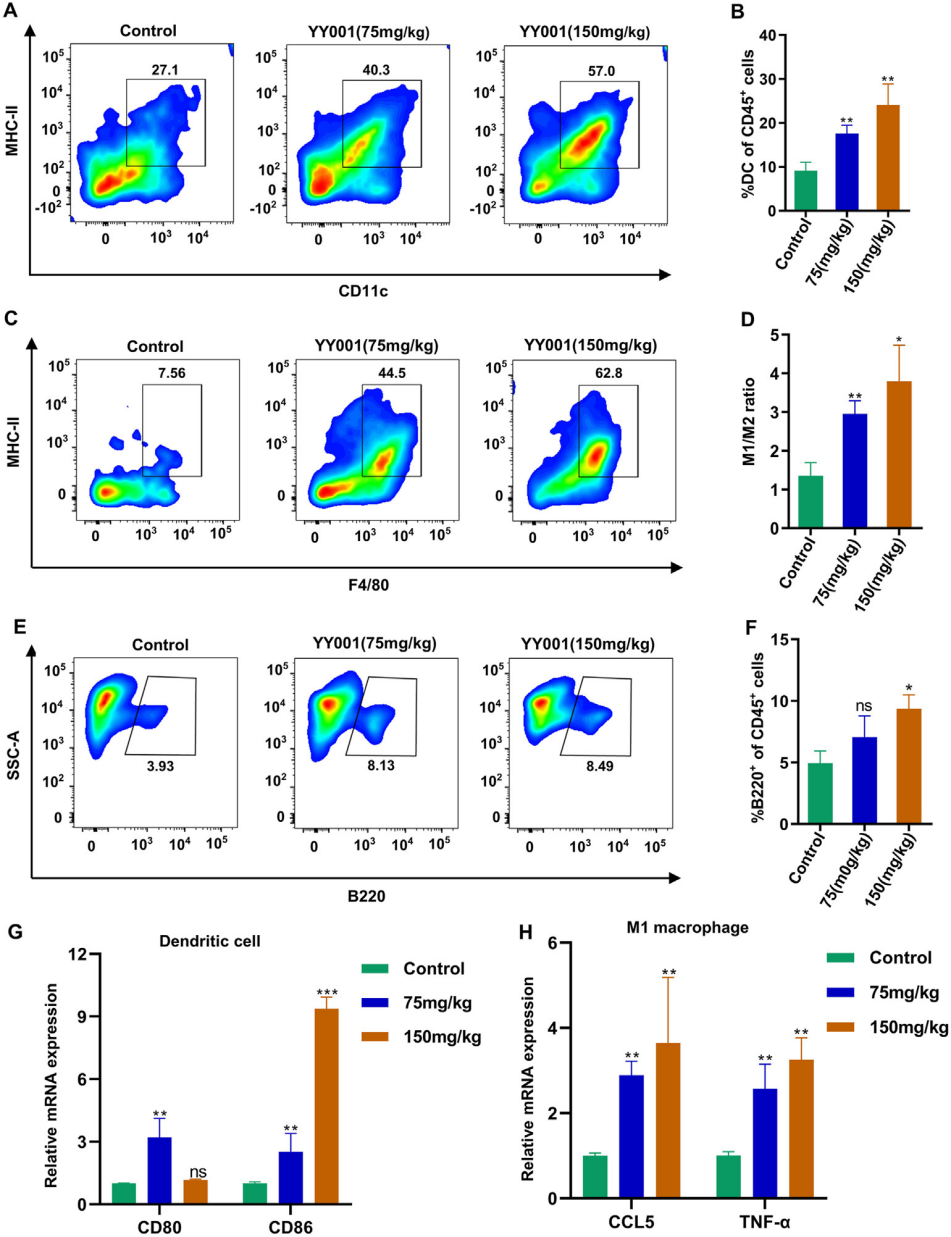
YY001 regulates the infiltration of antigen-presenting cells. **(A, B)** Dendritic cell (DC) infiltration in the tumor microenvironment by flow cytometry analysis. (A) Flow cytometry analysis. (B) Statistical result. **(C, D)** Flow cytometry analysis of macrophage infiltration in the tumor microenvironment. (C) Flow cytometry analysis. (D) Statistical graph of M1 macrophage to M2 macrophage ratio. **(E, F)** Flow cytometry analysis of B-cell infiltration in the tumor microenvironment. (E) Flow cytometry analysis. (F) Statistical result. **(G, H)** Expression of dendritic cells and M1 macrophages in tumor tissues was detected by quantitative PCR. ∗*P* < 0.05; ∗∗*P* < 0.01; ns, not significant.

Furthermore, antigen-presenting cells play a crucial role in T cell-mediated killing of cancer cells. Antigen-presenting cells communicate essential information about antigens to T cells, initiating T cell activation and promoting tumor-specific responses. This activation leads to the production of cytotoxic T cells that generate immunologically active materials to aid in tumor destruction. Consequently, the assessment was extended to antigen-presenting cells such as dendritic cells, M1 macrophages, and B cells. Flow cytometry analysis of tumor tissues revealed a significant increase in dendritic cells (Fig. 3A and B), M1 macrophages (Fig. 3C, D), and B cells (Fig. 3E and F) following YY001 treatment. Quantitative PCR analysis demonstrated elevated expression of both dendritic cells (Fig. 3G) and M1 macrophages (Fig. 3H) in tumor tissues. These results indicate that YY001 also promotes the infiltration of antigen-presenting cells into tumors, thereby stimulating an anti-tumor immune response in gastric cancer.

### YY001 regulates dendritic cell and MDSC differentiation

**Figure 4:**
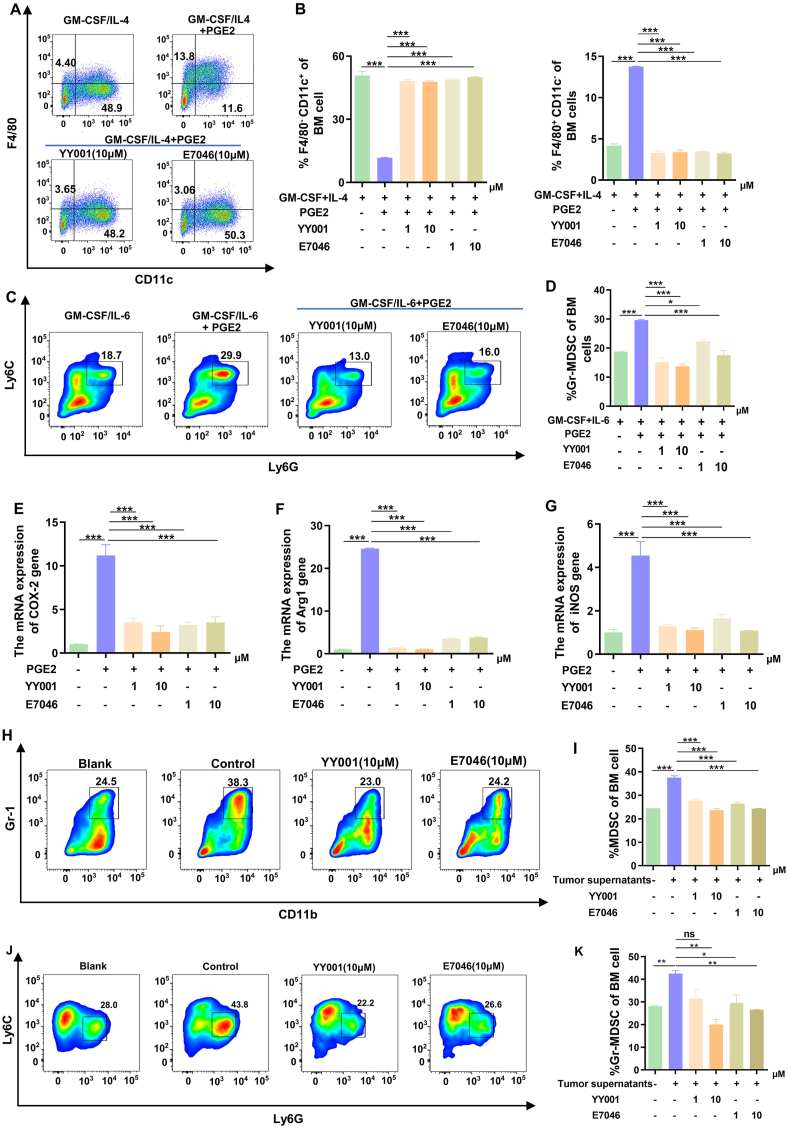
YY001 regulates the expression and polarization of dendritic cells (DCs) and MDSCs in gastric cancer. **(A)** Effect of prostaglandin E2 (PGE2) and EP4 antagonist on the differentiation of macrophages/dendritic cells in mouse bone marrow mononuclear cells detected by flow cytometry. **(B)** Analysis and statistics of flow cytometry results for macrophage and dendritic cell differentiation. **(C)** Flow cytometry to detect the effects of PGE2 and EP4 antagonists on the differentiation of mouse bone marrow-derived MDSCs. **(D)** Analysis and statistical results of flow analysis Granulocyte MDSCs (Gr-MDSCs). **(E–G)** Quantitative PCR to detect the effects of PGE2 as well as EP4 antagonists on the production of immunosuppressive molecules in MDSCs. **(H, I)** Flow cytometry analysis of the effect of tumor supernatants as well as EP4 antagonists on the differentiation of myeloid-derived MDSCs in mice. (H) Flow cytometry analysis result. (I) MDSC differentiation statistical result. **(J, K)** Flow cytometry analysis of tumor supernatant and EP4 antagonist on Gr-MDSCs. (J) Flow cytometry analysis result. (K) Statistical result. ∗*P* < 0.05; ∗∗*P* < 0.01; ∗∗∗*P* < 0.01; ns, not significant.

*In vivo* studies have shown that YY001 can activate the body's immune system and suppress tumors by modifying the tumor microenvironment. To investigate whether YY001 has the same effect *in vitro*, we conducted experiments to explore its role in the differentiation of bone marrow cells. Mouse bone marrow cells were isolated and cultured *in vitro* to study the effects of YY001. These cells were stimulated with granulocyte-macrophage colony-stimulating factor and IL-4, and the impact on cell differentiation was assessed by adding or not adding PGE2. Our findings indicate that PGE2 inhibits dendritic cell differentiation while promoting macrophage differentiation. However, even in the presence of PGE2, YY001 inhibited macrophage differentiation and restored dendritic cell differentiation (Fig. 4A and B). Furthermore, we induced the differentiation of mouse bone marrow cells into MDSCs using granulocyte-macrophage colony-stimulating factor and IL-6 *in vitro*. The presence of PGE2 significantly enhanced MDSC differentiation, but this differentiation was inhibited when treated with EP4 antagonists, even in the presence of PGE2 (Fig. 4C and D). We also examined the expression of MDSC markers using quantitative PCR and found that YY001 significantly reduced the expression of markers such as COX2, Arg1, and inducible nitric oxide synthase (Fig. 4E–G).

To further confirm the immunological effects of YY001, we cultured bone marrow cells *in vitro* using supernatant from gastric cancer cells. Flow cytometry analysis revealed that the proportion of bone marrow cells differentiating into MDSCs was higher in the tumor supernatant group compared with the control group (Fig. 4H and I). Further analysis showed that the tumor supernatant mainly induced an increased differentiation of granulocyte MDSCs (Fig. 4J and K). However, when YY001 was added to the tumor supernatant, the differentiation of MDSCs was effectively suppressed, and this inhibitory effect was more pronounced at higher concentrations (Fig. 4I, K). These results are consistent with our *in vivo* findings and demonstrate that YY001 can enhance immune function in gastric cancer.

### The combination of YY001 with apatinib resulted in a complete regression of MFC tumors

**Figure 5:**
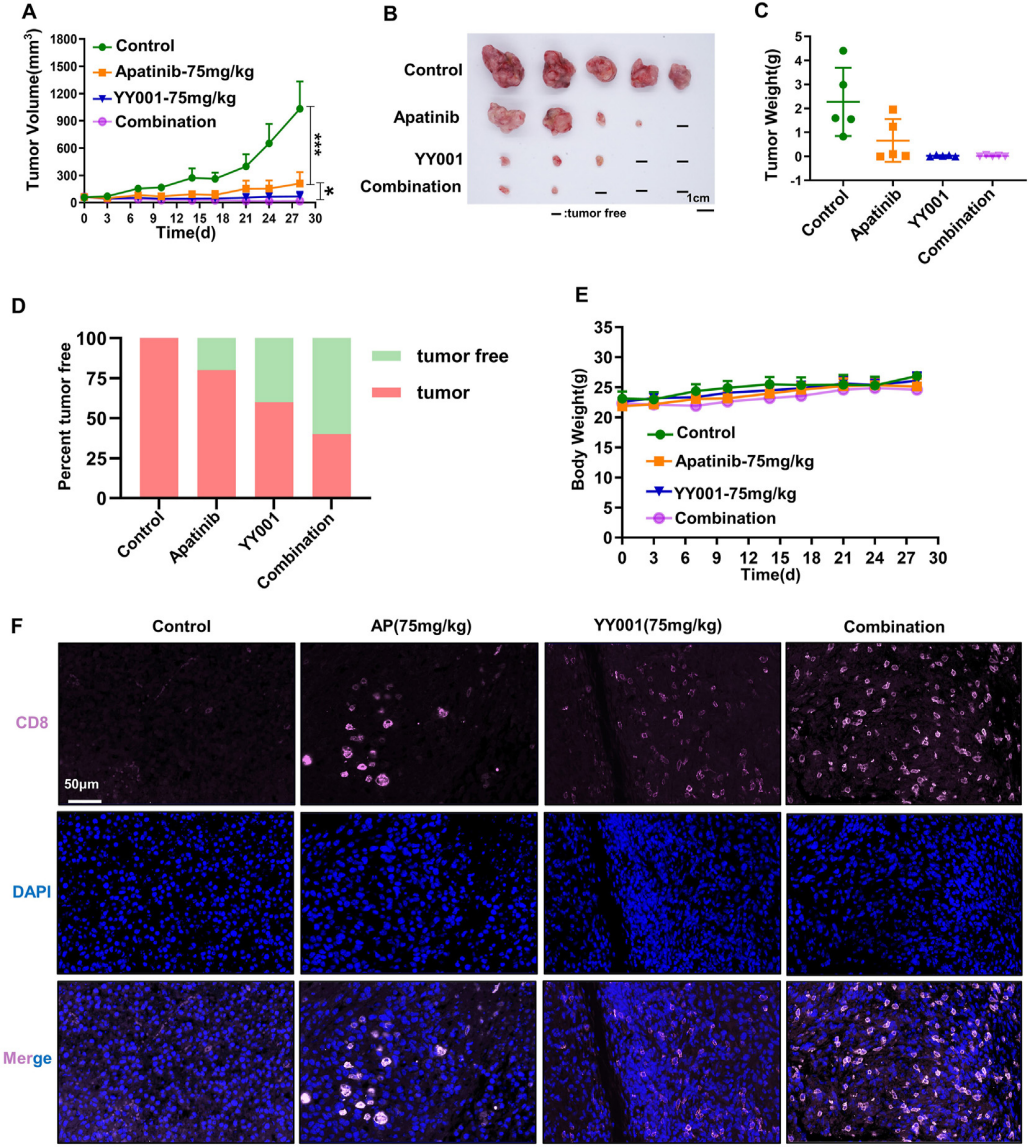
YY001 in combination with apatinib induces regression of gastric cancer and prolongs survival in mice. **(A–E)** MFC subcutaneous growth model for gastric cancer to assess the antitumor activity of YY001 in combination with apatinib. (A) Subcutaneous growth tumor growth curve of MFC gastric cancer. (B) Tumor peeling white light plot. (C) Peeling tumor weight statistics. (D) Tumor regression rate after treatment statistics. (E) Statistical curves of body weight changes in mice after drug treatment. **(F)** Detection of CD8^+^ T cell infiltration in the tumor microenvironment after single or combined therapy by immunofluorescence staining. ^∗^*P* < 0.05; ^∗∗^*P* < 0.01; ^∗∗∗^*P* < 0.01.

**Figure S2:**
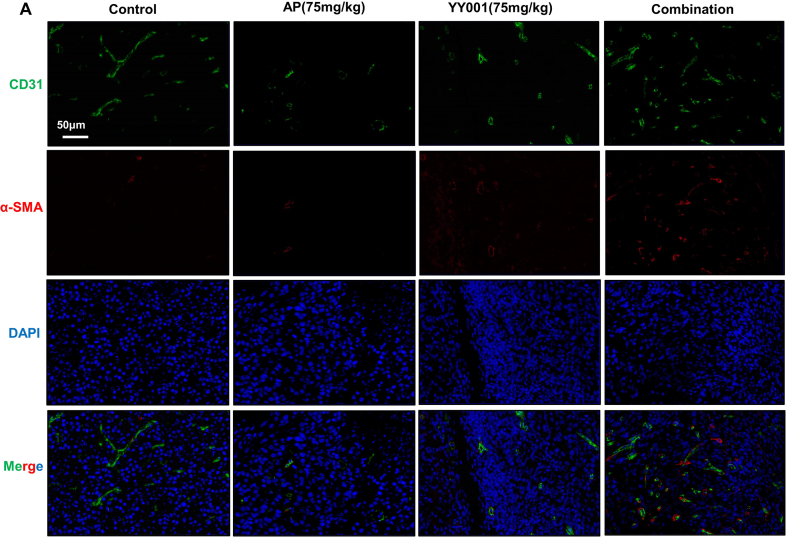


Apatinib, a powerful tyrosine kinase inhibitor that targets vascular endothelial growth factor receptor 2, has demonstrated its safety and effectiveness as a small-molecule anti-angiogenic drug for treating advanced gastric cancer patients who have not responded to standard chemotherapy.^42^^,^^43^ In light of this, we developed a treatment regimen combining YY001 with apatinib. Both the single drug and the combination drug significantly inhibit tumor growth when compared with the control group (Fig. 5A). Upon removing the tumor tissue, it becomes evident that the single drug treatment effectively eliminates a portion of the tumor (Fig. 5B), and the tumor weight is noticeably reduced compared with the control group (Fig. 5C). The tumor elimination rates for YY001 and apatinib are 40% and 20% respectively (Fig. 5D). However, there is a considerable inter-individual variation in the response to apatinib, with some cases exhibiting no response. When YY001 and apatinib are combined, tumor growth is almost completely inhibited, resulting in a 60% tumor elimination rate. Notably, no weight loss occurs during the administration of the combination therapy, indicating that the combination does not increase toxicity or intolerance (Fig. 5D, E). To further investigate the mechanism of tumor cell death induced by single or combination drugs, we analyzed the expression of CD8^+^ T cells through immunofluorescence analysis. The results demonstrate a significant increase in the infiltration of CD8^+^ T cells compared with the control (Fig. 5F). Immunofluorescence analysis of the angiogenic marker CD31 and the tumor-associated fibroblast marker α-SMA did not reveal significant changes (Fig. S2A). These findings suggest that YY001 and apatinib exhibit a synergistic effect in the treatment of gastric cancer.

### The combination of YY001 with PD-1 antibody resulted in complete regression of MFC tumors

**Figure 6:**
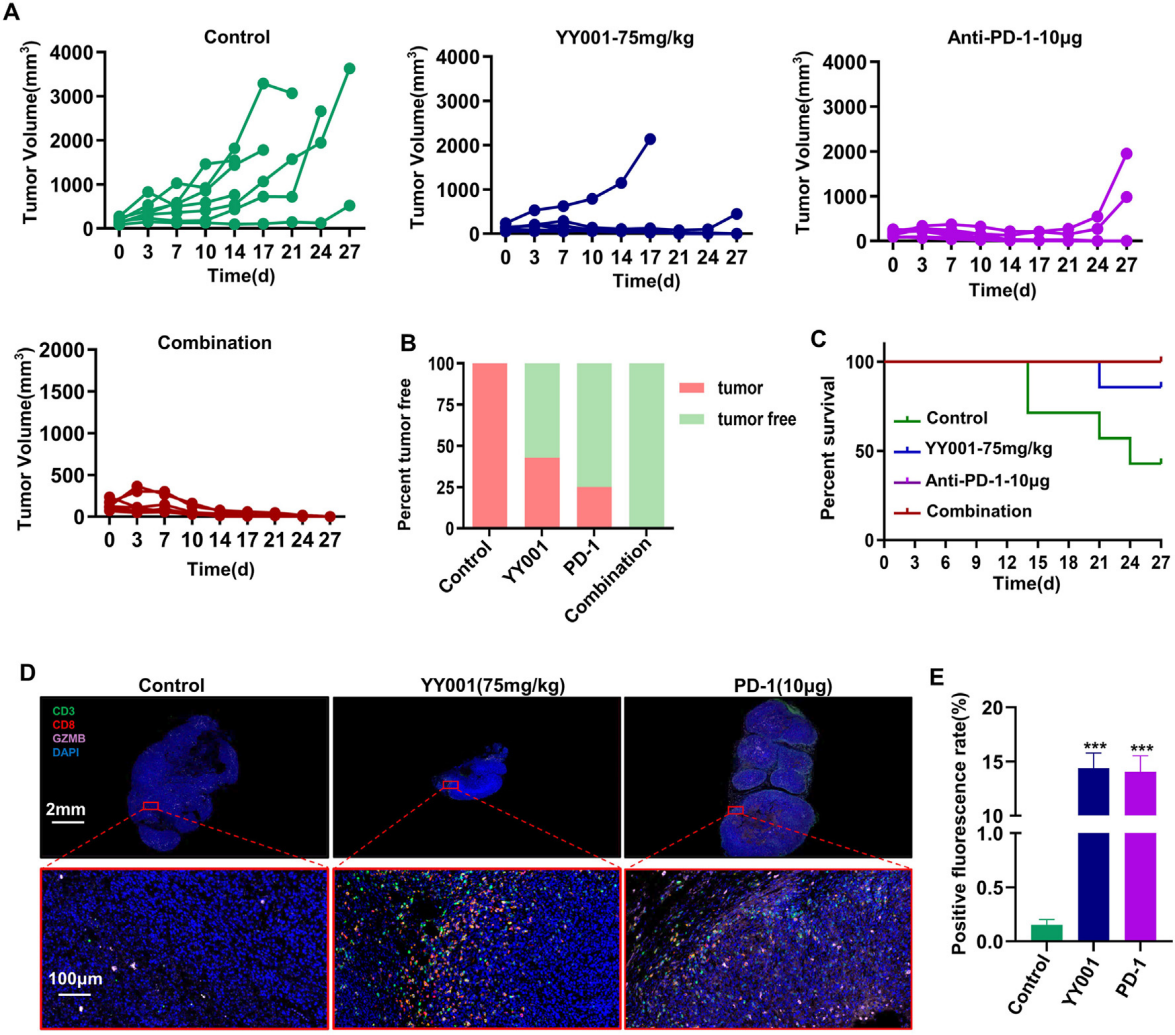
YY001 in combination with PD-1 antibody induces complete regression of gastric cancer tumors and prolongs survival of mice. **(A–C)** MFC gastric cancer in an immunocompetent mouse subcutaneous growth model to assess the combined anti-tumor activity of YY001 and PD-1 antibodies. (A) MFC subcutaneous tumor growth curve. (B) Tumor regression rate statistics. (C) Mouse survival curve. **(D)** Immunofluorescence detection of T-cell infiltration in tumor tissues. **(E)** Immunofluorescence detection statistics.

**Figure S3:**
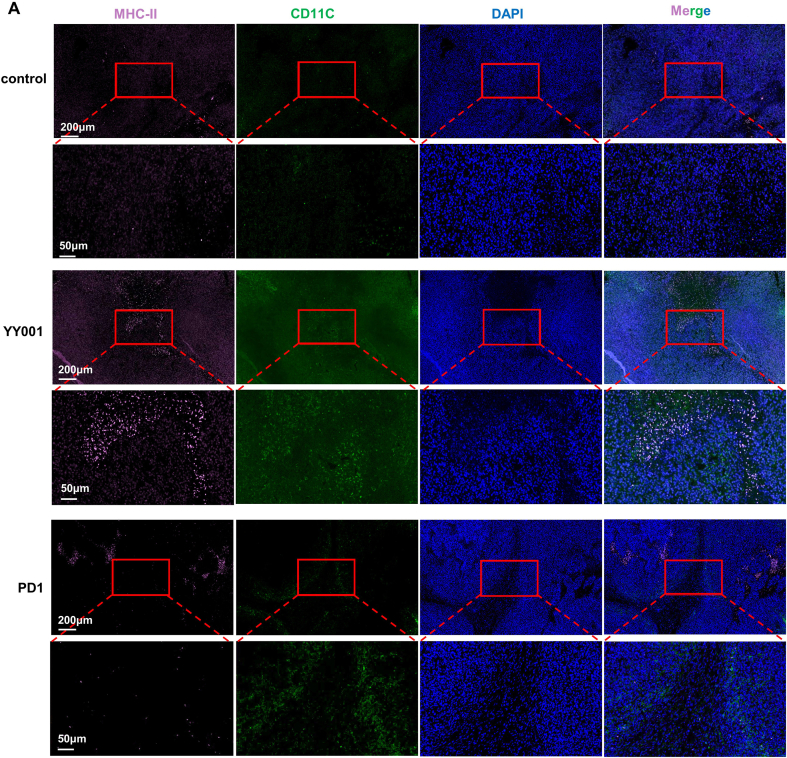


In recent years, immune checkpoint blockade therapy has been utilized in tumor treatment and has shown promising results. However, the response rates of immune checkpoint inhibitors are generally low. Previous studies have indicated that the PD-1 antibody did not significantly improve gastric cancer treatment.^44^^,^^45^ To address the issue of the low efficacy of PD-1 antibodies in gastric cancer treatment, we conducted a study to evaluate the effectiveness of combining YY001 treatment with PD-1 antibodies. In a subcutaneous tumor-bearing mouse model, the experiment results demonstrated that treatment with PD-1 antibodies alone or YY001 alone suppressed the growth of MFC tumors. Some of the tumors even completely disappeared after treatment (Fig. 6A). Moreover, when YY001 and PD-1 antibodies were used in combination, they effectively inhibited the growth of MFC tumors in mice and led to the complete elimination of the tumors (Fig. 6A, B). The combination therapy with YY001 and PD-1 antibodies significantly prolonged the survival of the mice (Fig. 6C). Immunofluorescence staining of tumor tissues after treatment with YY001 or PD-1 antibodies revealed a noticeable increase in the infiltration of CD3^+^ T cells and CD8^+^ T cells compared with the control group (Fig. 6D). Statistical analysis of the fluorescence results also revealed a significant increase in infiltration of T cells into the tumor after treatment (Fig. 6E). Additionally, we examined the expression of MHC-II and CD11C in tumor tissues using immunofluorescence, and the expression levels were found to be higher compared with the control group (Fig. S3A). These results demonstrate that the combination of YY001 and PD-1 antibodies has a synergistic effect in inhibiting gastric cancer, offering a promising therapeutic approach for its treatment.

### The combination of YY001 with 5-Fu became less effective in tumor inhibition in gastric cancer and YY001 has a limited effect on melanoma

**Figure 7:**
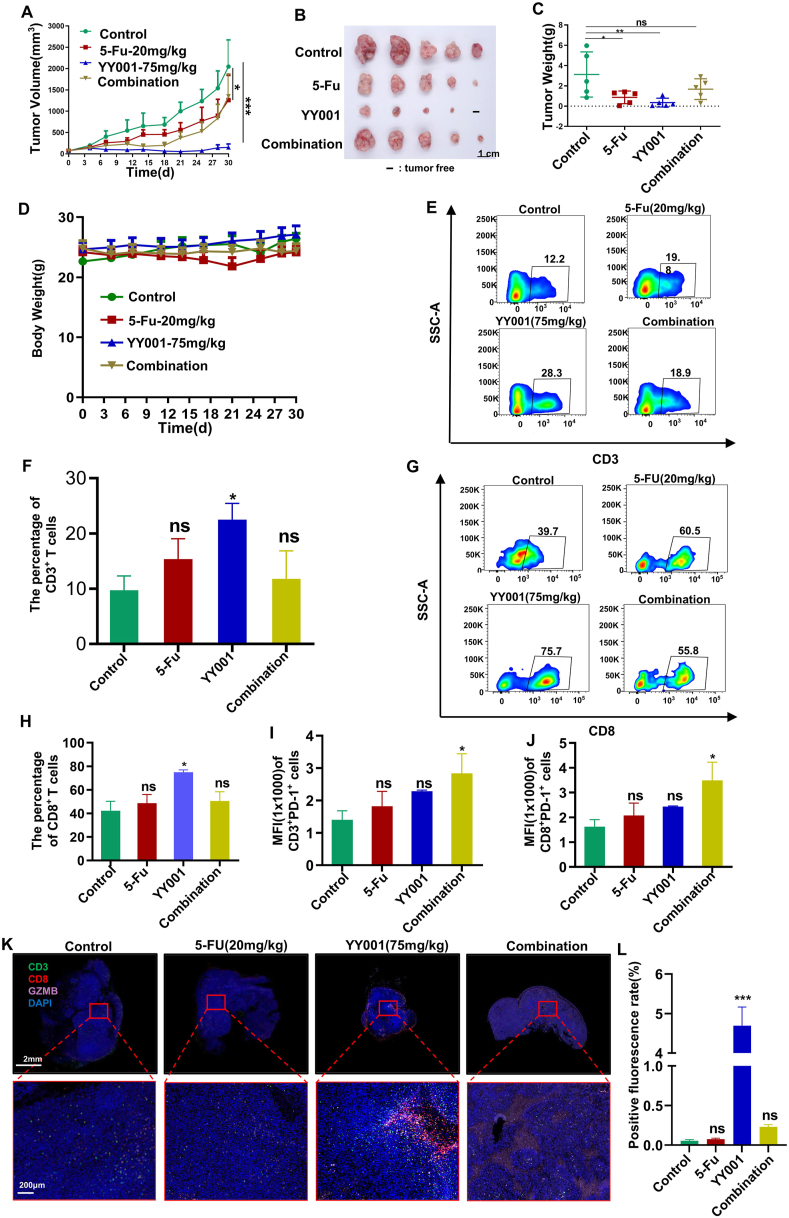
YY001 in combination with 5-Fu inhibits gastric cancer poorly. **(A–D)** MFC subcutaneous growth model of gastric cancer to assess the antitumor activity of YY001 in combination with 5-Fu. (A) Tumor growth curve. (B) Tumor peeling white light plot. (C) Peeling tumor weight statistics. (D) Mouse weight change plot. **(E–J)** Flow cytometry analysis of T cell infiltration in the tumor microenvironment. (E) CD3 cell flow analysis. (F) Statistical result of CD3 cell expression. (G) CD8 cell flow analysis. (H) Statistical result of CD8 cell expression. (I) PD-1 expression statistics on CD3 cells. (J) PD-1 expression statistics on CD8 cells. **(K)** Immunofluorescence detection of T-cell infiltration by 5-FU, YY001, and their combination. **(L)** Immunofluorescence detection statistics. ^∗^*P* < 0.05; ^∗∗^*P* < 0.01; ^∗∗∗^*P* < 0.01; ns, not significant.

As an antimetabolite, fluorouracil is commonly used as a first-line adjuvant therapy for patients with advanced gastric cancer.^46^ However, prolonged use of 5-Fluorouracil (5-Fu) can lead to drug resistance and cause intestinal inflammation. Therefore, we developed a combined protocol for the subcutaneous tumor model. Our observations showed that YY001 had a stronger inhibitory effect compared with 5-Fu, but its effectiveness decreased when used in combination (Fig. 7A–C). Regular measurements of body weight indicated that the combination treatment of YY001 and 5-Fu was well tolerated as the mice did not experience weight loss (Fig. 7D). We evaluated T-cell infiltration following combination therapy and found that there was no increase in T-cell infiltration in tumor tissue after combining 5-Fu with YY001, including CD3^+^ T cells (Fig. 7E, F) and CD8^+^ T cells (Fig. 7G, H). Additionally, there was an increased expression of PD-1 on T cells in tumor tissue (Fig. 7I, J). Immunofluorescence analysis was conducted to assess the infiltration of immune cells in 5-Fu, YY001, and the combination treatment, as well as the expression of GZMB, a molecule associated with T-cell function. The results demonstrated that T-cell infiltration in the tumor microenvironment was reduced after 5-Fu treatment, thereby attenuating T-cell function (Fig. 7K). Statistical analysis of the immunofluorescence results also indicates that 5-Fu weakens the effectiveness of YY001(Fig. 7L).

**Figure S4:**
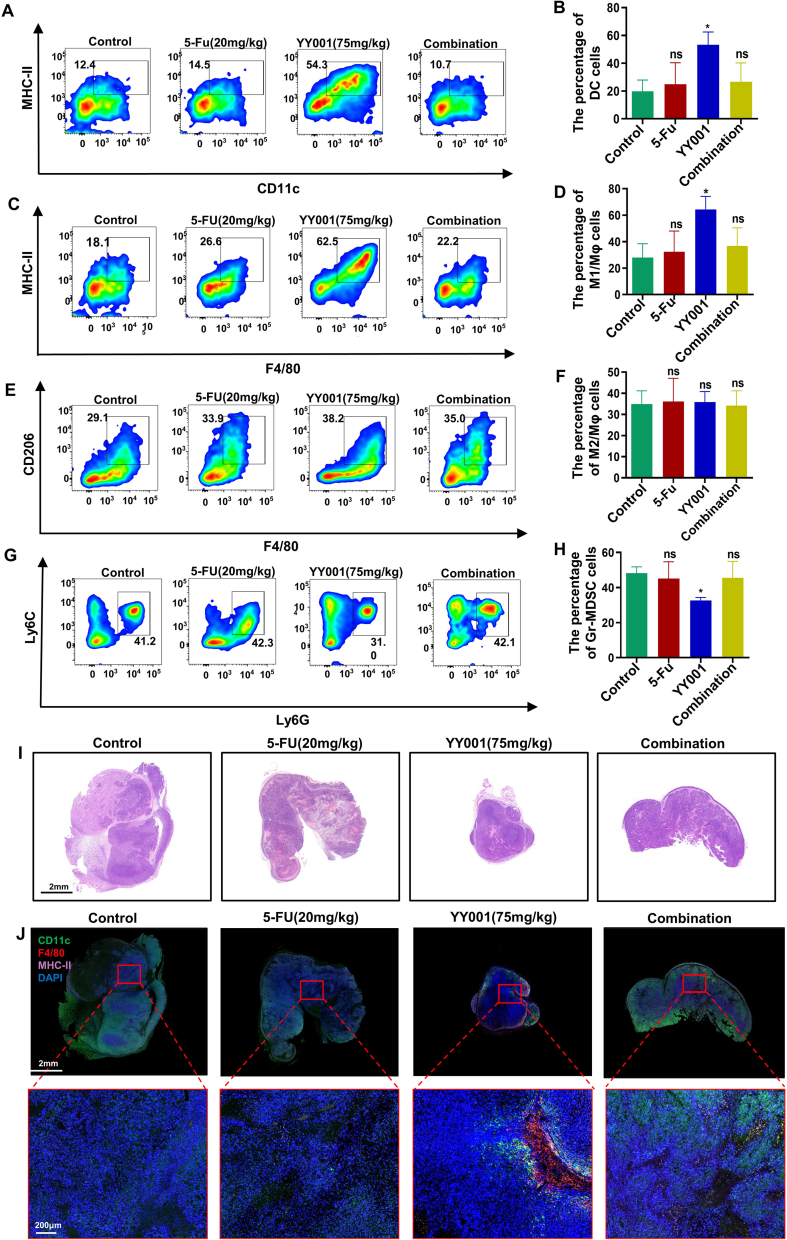


Furthermore, we performed flow cytometry analysis to investigate antigen-presenting cells in tumor tissues. We observed a decrease in dendritic cells when 5-Fu and YY001 were used in combination versus when YY001 was used alone (Fig. S4A, B). The polarization of M1 macrophages, which promote tumor immune function, was also reduced (Fig. S4C, D). However, there were no significant changes in M2 macrophages or granulocyte MDSCs after drug administration compared with the control group (Fig. S4E–H). These findings were consistent with the results obtained from hematoxylin and eosin staining of tumor tissues and immunofluorescence staining (Fig. S4I, J). Therefore, the experimental results suggest that 5-Fu may reduce T-cell infiltration in MFC tumors, preventing synergistic effects with YY001.

**Figure S5:**
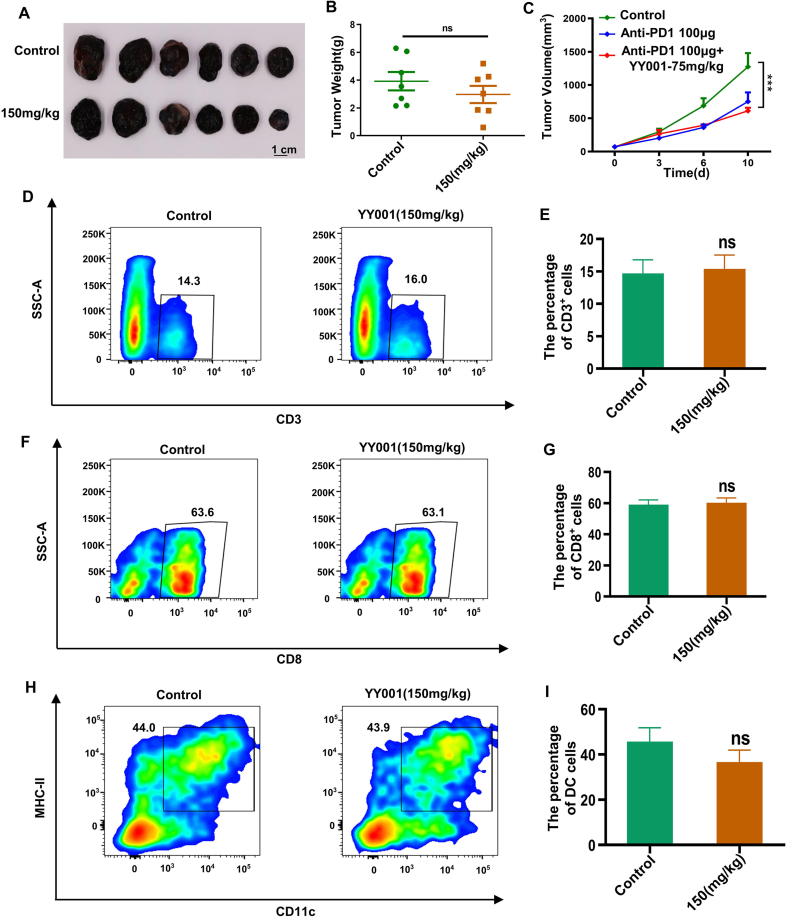


We further investigated the pre-constructed melanoma model (Fig. 1A). There was no significant difference observed in tumor tissue stripping and weighing compared with the control (Fig. S5A, B). Additionally, when YY001 was combined with PD-1 antibody, it did not enhance the effect of PD-1 antibody (Fig. S5C). Flow cytometry analysis was conducted to analyze the infiltration of T cells in the tumor tissue, and the results showed a slight increase in CD3^+^ T cell infiltration, but the difference was not statistically significant (Fig. S5D, E). CD8^+^ T cell infiltration also did not show a significant increase compared with the control (Fig. S5F, G), and the infiltration of dendritic cells with antigen-presenting effects did not exhibit a significant difference either (Fig. S5H, I).

To further examine the impact of YY001 on macrophage polarization in the treatment of melanoma, we performed a flow cytometry analysis. The results revealed no significant effect on M1 macrophages, which promote tumor immune function against tumors (Fig. S6A, B), or on M2 macrophages (Fig. S6C, D), which have an immunosuppressive effect, compared with the control group. Additionally, our analysis showed no significant change in the infiltration of myeloid-derived suppressor cells, including mononuclear MDSCs (Fig. S6E, F) and polymorphonuclear MDSCs (Fig. S6G, H), compared with the control group. These results indicate that YY001 may not affect the immune microenvironment remodeling of melanoma.

## Discussion

Tumors are complex systems, and cancer cells employ various mechanisms to create a favorable environment for growth and spread. They rely on immune cells, fibroblasts, vascular cells, tumor-associated macrophages, and MDSCs to create a robust tumor-suppressive microenvironment that supports tumor growth, metastasis, and escape.^47^ In this study, we targeted the PGE2-EP4 signaling pathway to block the therapeutic effect of immunosuppression on gastric cancer. Through our search, we identified a novel selective EP4 antagonist called YY001, which can remodel the tumor microenvironment and block immunosuppression. Additionally, YY001 demonstrated tumor suppressive effects in various cancer models and enhanced the infiltration of cytotoxic T cells and antigen-presenting cells in gastric cancer tumor tissues.

PGE2 is a key factor in gastrointestinal tumors, and COX2 catalyzes the biosynthesis of prostaglandins, which are overexpressed in cancers such as colorectal cancer.^48^ In the PGE2-EP4 signaling pathway, EP4 serves as a potential anti-tumor target, and excessive activation of this pathway damages dendritic cells and natural killer cells, creating an immunosuppressive environment. Clinical trials have shown that E7064, an EP4 antagonist, exhibits good tolerability and achieves favorable therapeutic effects when used alone or in combination with chemotherapy drugs.^49^ Our findings suggest that YY001 can modulate the immune response against gastric cancer tumors by reducing immunosuppression and increasing the infiltration of antigen-presenting cells in tumor tissues.

Studies indicate that EP4 plays a crucial role in the function and differentiation of human and murine-derived myeloid cells.^50^ EP4 also acts as an important receptor for prostaglandins in human and murine macrophages.^51^^,^^52^ The tumor microenvironment lacks T cell infiltration, while immunosuppressive cells, including tumor-associated macrophages, MDSCs, and regulatory T cells, contribute to the immunosuppressive microenvironment.^47^ EP4 is involved in the depletion of cytotoxic T cells and the differentiation of myeloid cells.^53^ Our study demonstrated that tumor-associated macrophages and MDSCs were the target cells of YY001, and their numbers were significantly reduced in treated gastric cancer tumor tissue. However, further research is needed to understand how YY001 regulates the role of these immune cells in the microenvironment.

Immunosuppressive cell populations, such as regulatory T cells and MDSCs, are present in the tumor immune microenvironment, which reduces the infiltration of cytotoxic T cells. Additionally, tumor cells up-regulate PD-L1 expression, enabling them to evade killing by cytotoxic T cells. Blocking the PD-1 pathway using monoclonal antibodies enhances tumor recognition by the immune system and facilitates the development of immune checkpoint inhibitors. However, immune checkpoint blockade therapy has shown a weak response in gastric cancer. Research has shown that PD-1 expression is positively regulated by PGE2-EP4 signaling, leading to immune suppression.^54^ Our results revealed that a combination of YY001 and PD-1 antibodies completely inhibited gastric cancer growth and resulted in successful tumor elimination. This combined treatment increased T cell infiltration in tumor tissue, reshaping the immune microenvironment and achieving the goal of inhibiting tumor growth and eliminating tumors. The complete inhibition of MFC gastric cancer growth and tumor elimination by the combination of YY001 and PD-1 antibodies deserve further in-depth study.

Our animal model results demonstrate that YY001 is more effective in gastric cancer than in other tumors, particularly melanoma. Analysis of proteins related to the COX-2/PGE2/EP4 pathway showed high expression of COX-2 in gastric cancer cells but not in melanoma cells. This difference in expression may be related to the degree of activation and involvement of the COX-2/PGE2/EP4 pathway, but further research is necessary to understand the underlying mechanisms. Combining YY001 with PD-1 antibodies and apatinib has shown promising results and may have potential clinical implications that warrant further investigation. However, it should be noted that YY001 has the opposite effect when combined with 5-Fu, possibly due to the impact of 5-Fu on the proliferation and infiltration of T cells in the tumor microenvironment, thereby weakening the anti-tumor immune potential.^55^ These findings provide valuable guidance for clinical applications and emphasize the need for further in-depth studies.

**Figure 8:**
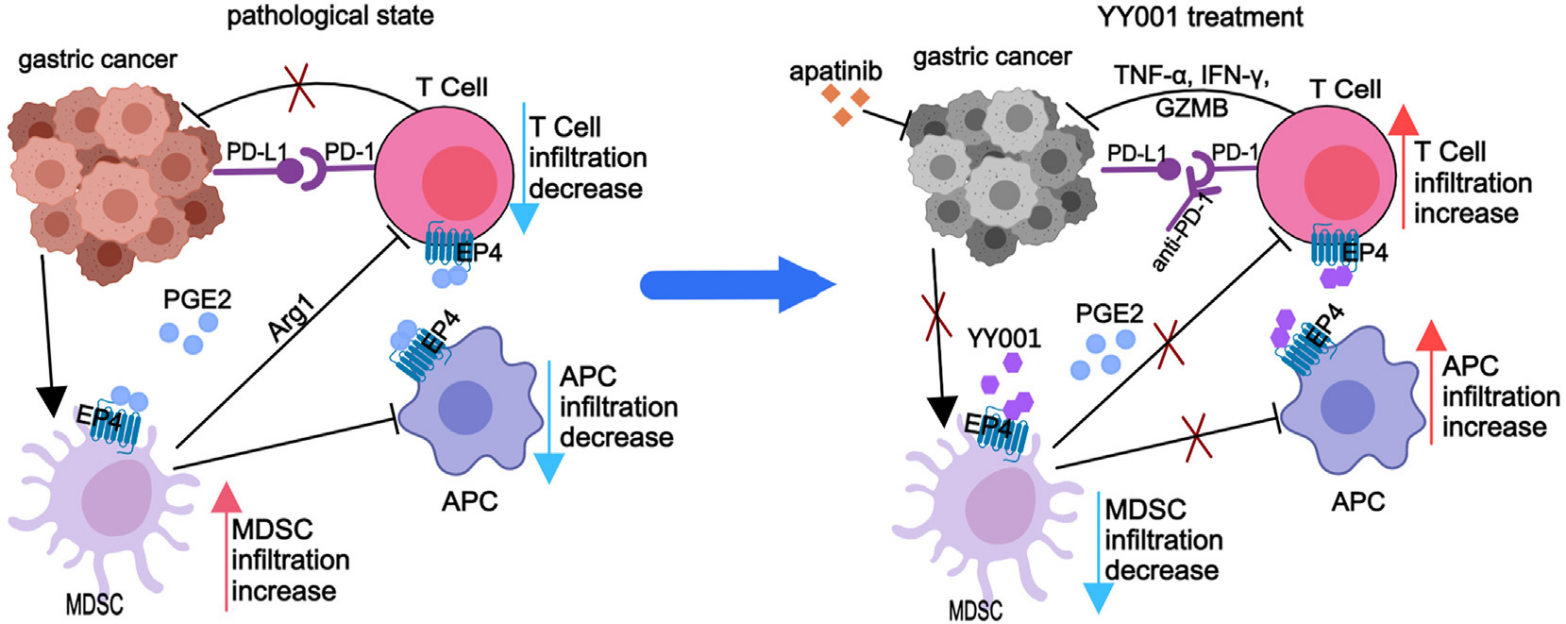
Schematic diagram of YY001 for gastric cancer inhibition. In the gastric cancer tumor microenvironment, gastric cancer cells promote the infiltration of MDSCs. PGE2 further activates MDSCs and induces the secretion of inhibitory cytokines such as Arg1, leading to inhibition of antigen-presenting cells and T cell infiltration and function, creating an immunosuppressive microenvironment. When treated with YY001, MDSC infiltration is inhibited, while antigen-presenting cells and T cell infiltration are increased, reactivating the body's immune response. The combination of YY001 with apatinib or PD-1 antibodies synergistically inhibits gastric cancer. This figure was created using MedPeer (www.medpeer.cn).

In conclusion, our study demonstrates that YY001, an EP4 antagonist, can reshape the tumor microenvironment in gastric cancer and reactivate the body's immune response (Fig. 8), leading to anti-tumor effects. This research provides a novel method and establishes a research foundation for the treatment of gastric cancer.

## Ethics declaration

The Animal Center of East China Normal University provided all the animals, and their welfare was monitored in compliance with the guidelines of the Animal Investigation Committee at the Institute of Biomedical Sciences, East China Normal University.

## Author contributions

Mengmeng Guo: Investigation, methodology, writing-original draft, writing-editing. Pang Hu: Methodology, investigation, writing-original draft. Jiayi Xie: Data curation, form analysis. Kefu Tang: Conceptualization, methodology. Shixiu Hu: Methodology. Jialiang Sun: Methodology, formal analysis. Yundong He: Methodology. Jing Li: Methodology. Weiqiang Lu: Methodology. Huirong Liu: Formal analysis, conceptualization. Mingyao Liu: Conceptualization, funding acquisition, project administration Zhengfang Yi: Investigation, funding acquisition, conceptualization, writing-review and editing, project administration. Shihong Peng: Methodology, funding acquisition, conceptualization, writing-review and editing, project administration.

## Conflict of interests

The authors of this article have no conflicting interests.

## Funding

This study was supported by the 10.13039/501100001809National Natural Science Foundation of China (No. 82073310 to Z. Yi; 81830083 to M. Liu; 81802970 to S.P.), the National Key R&D Program of China (No. 2018YFA0507001 to M. Liu), The Science and Technology Commission of Shanghai Municipality, China (No. 20JC1417900 to Z. Yi; 22QB1405600 to S. P), ECNU Construction Fund of Innovation and Entrepreneurship Laboratory (Shanghai, China) (No. 44400-20201-532300/021 to Z. Yi), and ECNU Public Platform for innovation (Shanghai, China) (011 to S. P), and Pujiang Scholar Program Award (Shanghai, China) (No. 22PJ1402700 to Y. H).

## Figures and Tables

**Figure S6:**