# Supplementary material for: Remodeling the immune microenvironment for gastric cancer therapy through antagonism of prostaglandin E2 receptor 4
Source: Genes Dis. 2023 Nov 10;11(4):101164. doi: 10.1016/j.gendis.2023.101164 (PMC10980949; doi:10.1016/j.gendis.2023.101164)
Supplement: Multimedia component 1 [file mmc1.docx]

**Supplementary information**

**Supplementary methods**

**Western blot analysis**

Cells and tumor tissues were lysed with RIPA buffer containing protease inhibitors and phosphatase inhibitors. The normalized total proteins were separated in 10% SDS polyacrylamide gels. The protein is transferred from the gel to a nitrocellulose membrane, closed and incubated with primary antibody overnight at 4°C. The membranes were washed and incubated with the secondary antibody for 1 hour at room temperature and the signal was detected using the Infrared Imaging System (LI-COR Biosciences, Lincoln, NE).

**Transwell assay**

24-well plates were inserted with Transwell with 8 μM pores. 1×10^5^ cells were plated in the upper Transwell chamber with RPMI1640, and the lower chamber was filled with 500 μL of complete medium. The cells were treated with varying concentrations of YY001 for 24 hours. After paraformaldehyde fixation, the cells on the upper side of the filter membrane were gently wiped off with a cotton swab. Stained with 0.1% crystalline violet and imaged under the microscope.

**Supplementary Tables:**

**Table S1. The sequence of Real-time PCR primers.**

| **Gene name** | **Forward primer** | **Reverse primer** |
| --- | --- | --- |
| Mouse CXCL9 | GCAGTGTGGAGTTCGAGGAA | AGTCCGGATCTAGGCAGGTT |
| Mouse CXCL10 | CCAAGTGCTGCCGTCATTTTC | GGCTCGCAGGGATGATTTCAA |
| Mouse CXCL11 | TATGTTCAAACAGGGGCGCT | TCCAGGCACCTTTGTCGTTT |
| Mouse TNF-α | CCTGTAGCCCACGTCGTAG | GGGAGTAGACAAGGTACAACCC |
| Mouse GZMB | TGTGAAGCCAGGAGATGTGTGCTA | TCAGCTCAACCTCTTGTAGCGTGT |
| Mouse perforin | CCGCATCTCGGTCCTTAC | TCGGGTTCTGTTCTTCCA |
| Mouse IFN-γ | TGAACGCTACACACTGCATCTTG | TGGCAGTAACAGCCAGAAACAG |
| Mouse Arg1 | TGGCTTGCGAGACGTAGAC | GCTCAGGTGAATCGGCCTTTT |
| Mouse  COX2 | TCAAAAGAAGTGCTGGAAAAGGTT | TCTACCTGAGTATCTTTGACTGTG |
| Mouse iNOS | GTTCTCAGCCCAACAATACAAGA | GTGGACGGGTCGATGTCAC |

**Table S2. List of antibodies**

| **Antibodies** | **Source** | **Identifier** |
| --- | --- | --- |
| APC anti-mouse CD3 | BioLegend | 100236 |
| APC anti-mouse CD8a | BioLegend | 100712 |
| FITC anti-mouse/human CD11b antibody | BioLegend | 101206 |
| APC anti-mouse Ly-6G/Ly-6C (Gr-1) | BioLegend | 108412 |
| APC anti-mouse F4/80 antibody | BioLegend | 123137 |
| PE anti-mouse CD279 (PD-1) | BioLegend | 135205 |
| Alexa Fluor® 700 Rat Anti-Mouse CD45 | BD Pharmingen™ | 560510 |
| BV605 Hamster Anti-Mouse CD11c | BioLegend | 117334 |
| Anti-CD206 (MMR) Mouse PE FCAb | BioLegend | 141706 |
| COX-2 | CST | 12282 |
| PD-L1 | CST | 13684 |
| GAPDH | CST | 5174 |

**Figure S1** YY001 does not inhibit the growth and migration of gastric cancer cells. **(A)** Western blot detection of COX2 and PD-1 protein expression in CT26, MFC, and other cell lines. **(B)** Determination of cell viability of mouse gastric cancer cells after drug treatment. **(C)** Determination of cytotoxicity of YY001 on different gastric cancer cells. **(D)** The effect of YY001 on the migratory effect of mouse gastric cancer cells as shown by Transwell tests.

**Figure S2** No significant change in angiogenesis. **(A)** Immunofluorescence detection of CD31, α-SMA monotherapy, and changes after combination therapy.

**Figure S3** Increased expression of antigen-presenting cells. **(A)** MHC-II and CD11C immunofluorescence staining.

**Figure S4** Modulation of antigen-presenting cells and MDSCs by single use of 5-Fu or YY001 or their combination. **(A-B)** Flow cytometry counts to detect changes in dendritic cells in tumor tissues. **(C-D)** Flow cytometry counts to detect changes in M1 macrophages in tumor tissue. **(E-F)** Flow cytometry counts to detect changes in M2 macrophages in tumor tissue. **(G-H)** Flow cytometric detection of granulocyte MDSC (Gr-MDSC) changes in tumor tissues. **(I)** Hematoxylin and eosin staining of tumor tissues. **(J)** Immunofluorescence staining of tumor tissues. ^*^*P* < 0.05; ns, not significant.

**Figure** **S5** YY001 has a limited effect on melanoma. **(A–C)** YY001 has a weak inhibitory effect on the growth of melanoma. (A) Tumor peeling white light photo. (B) Tumor weight statistics. (C) Melanoma growth curve. **(D–G)** Effect of YY001 on T-cell infiltration in melanoma. (D) Flow analysis of CD3^+^ T cells. (E) Statistical analysis of CD3^+^ T cell expression. (F) Flow analysis of CD8^+^ T cells. (G) Statistical result of CD8^+^ T cell expression. **(H, I)** Infiltration of dendritic cells (DCs) in the tumor microenvironment by flow analysis. (H) Flow analysis of DCs. (I) Statistical result of flow analysis. ^***^*P* < 0.001; ns, not significant.

**Figure S6** Effect of YY001 on immunosuppressive cells in melanoma. **(A–D)** Flow cytometry analysis of the regulation of macrophage infiltration in melanoma by YY001. (A) Flow cytometry analysis of M1 macrophages. (B) Statistical result. (C) Flow cytometry analysis result of M2 macrophages. (D) Statistical result. **(E–H)** Flow cytometry analysis of the regulation of MDSC infiltration by YY001 in melanoma. (E) Flow cytometry analysis of mononuclear MDSC (MO-MDSC) polarization in melanoma. (F) Statistical results of MO-MDSC polarization. (G) Flow cytometry analysis results of polymorphonuclear (PMN)-MDSCs. (H) Statistics of flow analysis results. ns, not significant.
